# Multi-Center in-Depth Screening of Neonatal Deafness Genes: Zhejiang, China

**DOI:** 10.3389/fgene.2021.637096

**Published:** 2021-07-02

**Authors:** Luhang Cai, Ya Liu, Yaping Xu, Hang Yang, Lihui Lv, Yang Li, Qiongqiong Chen, Xiaojiang Lin, Yihui Yang, Guangwei Hu, Guofeng Zheng, Jing Zhou, Qiyong Qian, Mei-ai Xu, Jin Fang, Jianjun Ding, Wei Chen, Jiong Gao

**Affiliations:** ^1^Department of Otorhinolaryngology, The First Affiliated Hospital, Zhejiang University School of Medicine, Hangzhou, China; ^2^Department of Otorhinolaryngology, Jiangshan People’s Hospital, Quzhou, China; ^3^Department of Otorhinolaryngology, Fenghua People’s Hospital, Ningbo, China; ^4^Department of Obstetrics, The First Affiliated Hospital, Zhejiang University School of Medicine, Hangzhou, China; ^5^Department of Otorhinolaryngology, Kaihua People’s Hospital, Quzhou, China; ^6^Department of Otorhinolaryngology, Ningbo Women and Children’s Hospital, Ningbo, China; ^7^Department of Otorhinolaryngology, Zhoushan Hospital, Zhoushan, China; ^8^Department of Otorhinolaryngology, Shaoxing Second Hospital, Shaoxing, China; ^9^Department of Otorhinolaryngology, Ruian People’s Hospital, Wenzhou, China; ^10^Department of Otorhinolaryngology, Shengzhou People’s Hospital, Shaoxing, China; ^11^Department of Otorhinolaryngology, Sanmen People’s Hospital, Taizhou, China; ^12^Department of Otorhinolaryngology, Zhejiang Xin’an International Hospital, Jiaxing, China; ^13^Department of Otorhinolaryngology, Linhai First People’s Hospital, Taizhou, China; ^14^Beijing Genomics Institute, Shenzhen, China

**Keywords:** deafness, hearing screening, genetic screening, genetic deafness, newborn deafness

## Abstract

**Purpose:**

The conventional genetic screening for deafness involves 9–20 variants from four genes. This study expands screening to analyze the mutation types and frequency of hereditary deafness genes in Zhejiang, China, and explore the significance of in-depth deafness genetic screening in newborns.

**Methods:**

This was a multi-centre study conducted in 5,120 newborns from 12 major hospitals in the East-West (including mountains and islands) of Zhejiang Province. Concurrent hearing and genetic screening was performed. For genetic testing, 159 variants of 22 genes were screened, including *CDH23*, *COL11A1*, *DFNA5*, *DFNB59*, *DSPP*, *GJB2*, *GJB3*, *KCNJ10*, *MT-RNR1*, *MT-TL1*, *MT-TS1*, *MYO15A*, *MYO7A*, *OTOF*, *PCDH15*, *SLC26A4*, *SOX10*, *TCOF1*, *TMC1*, *USH1G*, *WFS1*, and *WHRN* using next-generation sequencing. Newborns who failed to have genetic mutations or hearing screening were diagnosed audiologically at the age of 6 months.

**Results:**

A total of 4,893 newborns (95.57%) have passed the initial hearing screening, and 7 (0.14%) have failed in repeated screening. Of these, 446 (8.71%) newborns carried at least one genetic deafness-associated variant. High-risk pathogenic variants were found in 11 newborns (0.21%) (nine homozygotes and two compound heterozygotes), and eight of these infants have passed the hearing screening. The frequency of mutations in *GJB2*, *GJB3*, *SLC26A4*, *12SrRNA*, and *TMC1* was 5.43%, 0.59%, 1.91%, 0.98%, and 0.02%, respectively. The positive rate of in-depth screening was significantly increased when compared with 20 variants in four genes of traditional testing, wherein *GJB2* was increased by 97.2%, *SLC26A4* by 21% and *MT-RNR1* by 150%. The most common mutation variants were GJB2c.235delC and SLC26A4c.919-2A > G, followed by GJB2c.299_300delAT. Homoplasmic mutation in *MT-RNR1* was the most common, including m.1555A > G, m.961T > C, m.1095T > C. All these infants have passed routine hearing screening. The positive rate of *MT-RNR1* mutation was significantly higher in newborns with high-risk factors of maternal pregnancy.

**Conclusion:**

The positive rate of deafness gene mutations in the Zhejiang region is higher than that of the database, mainly in GJB2c.235delC, SLC26A4 c.919-2A > G, and m.1555A > G variants. The expanded genetic screening in the detection rate of diseasecausing variants was significantly improved. It is helpful in identifying high-risk children for follow-up intervention.

## Introduction

Deafness is one of the most common birth defects in humans, often causing lifelong disability and seriously affecting the quality of life of individuals. According to WHO in 2018, more than 5% of the world’s total population (466 million) suffer from some form of disabling hearing loss and 34 million of these are children. 1.1 out of every 1,000 newborns suffer from hearing impairment (Butcher et al., 2019). It is estimated that over 900 million people will have disabling hearing loss by 2050 (World Health Organization, 2018). According to the *2012 China Birth Defect Prevention Report*, hearing impairment has become the second largest birth defect in China, with an incidence rate of 1–3‰ (Qin and Zhu, 2013), and about 30,000 newborns with severe hearing impairment are born every year (Xin et al., 2013). Therefore, it is important to carry out genetic screening for deafness in newborns, as this assists in detecting it as soon as possible and provides suitable intervention in time.

As early as 2019, Shearer et al. (2019) recommended deafness gene testing and believed that in the next decade, genome sequencing will become a supplement to universal physiological neonatal hearing screening. Currently, the combined screening of newborn hearing and deafness genes aid in identifying hearing loss at birth, give early warning to delayed, progressive, and ototoxic-drug deafness, and effectively avoid the occurrence of hearing loss. However, the vast majority of deafness gene screening is aimed only at hot spot variants of few genes. Although these genes and loci cover most of the deafness gene pathogenic and likely pathogenic variants in individuals, there is still a high proportion of omissions. In some areas of China, large-scale neonatal gene screening has been conducted (Wang et al., 2011, 2019; Dai et al., 2019), while a large-scale multi-center neonatal genetic screening or exact epidemiological survey on the incidence of deaf children in Zhejiang Province, which has been a major economic province along the southeast coast of China, has not been carried out. This study conducted newborn hearing and genetic screening in 12 major hospitals and delivery institutions, including the mountainous areas and Zhoushan Archipelago of the Zhejiang region. A large number of previous studies have also focused on four genes (*GJB2, GJB3, SLC26A, MT-RNR1*) and 20 variants (*GJB2*:c.35delG, c.167delT, c.176_191del16, c.235delC, c.299_300delAT; *GJB3*:c.538C > T, c.547G > A; *SLC26A*:c.281C > T, c.589G > A, c.919-2A > G, c.1174A > T, c.1226G > A, c.1229C > T, c.1707 + 5G > A, c.1975G > C, c.2027T > A, c.2162C > T, c.2168A > G; *MT-RNR1*:m.1494C > T, m.1555A > G). This study conducted expanded screening of 159 variants of 22 deafness genes. We comprehensively analyzed the genotypes, carrying spectrum, and frequency of deafness pathogenic/likely pathogenic variants in newborns of Zhejiang province, hoping to establish a database of disease-causing variants with regional characteristics to lay a foundation for the establishment of a database of deafness genes in China.

## Materials and Methods

### Subjects

The study was a prospective and multi-center study of 5,120 newborns from maternal and children’s health and general hospitals (including mountains and islands) in Zhejiang Province from January 2018 to December 2019. A total of 12 regional hospitals included Hangzhou, Fenghua, Jiaxing, Kaihua, Jiangshan, Linhai, Ningbo, Ruian, Sanmen, Shaoxing, Zhoushan, and Shengzhou. Medical history and physical examination of the participating subjects were collected, including detailed family history, drug exposure history, and potential deafness factors.

This study was carried out with the authorization of the Hospital Ethics Committee of The First Affiliated Hospital, Zhejiang University School of Medicine. The participating subjects signed informed consent forms before sample collection and agreed that the sequencing data could be used for research after anonymization.

### Blood Sample Collection

At least three dried blood spots were collected from the heels of the newborns on the third day after birth. When the samples were sent to the laboratory, the appearance and information of the samples were checked, and then into the laboratory information management system for establishing a unified blood card, questionnaire, and information file.

### Newborn Hearing Screening

Initial screening was performed using transiently evoked otoacoustic emission (TEOAE) testing. TEOAE was performed 48 h after birth. For those referred (hearing screening failed) after initial testing, a repeat TEOAE test was performed by the age of 42 days and diagnosed audiologically at the age of 3 and 6 months.

### Deafness Genetic Screening

Deafness genetic screening was used to identify 159 variants in 22 genes, including *CDH23*, *COL11A1*, *DFNA5*, *DFNB59*, *DSPP*, *GJB2*, *GJB3*, *KCNJ10*, *MT-RNR1*, *MT-TL1*, *MT-TS1*, *MYO15A*, *MYO7A*, *OTOF*, *PCDH15*, *SLC26A4*, *SOX10*, *TCOF1*, *TMC1*, *USH1G*, *WFS1*, and *WHRN*.

### Genetic Testing

Genomic DNA was extracted from the blood samples using the Nucleic Acid Purification kit (BGI, Shenzhen, China). Multiplex PCR was used to amplify the target sequence of enriched human genomic DNA and introduce the sample tag sequence for sample identification. After purification and physical interruption, the PCR products were flattened at the end and added “A” at the 3′ end to complete the connection of the special connector at the two ends of the PCR product under the action of DNA polymerase, ligase, and phosphatase, and the preparation of the library was completed by magnetic bead purification.

**FIGURE 1:**
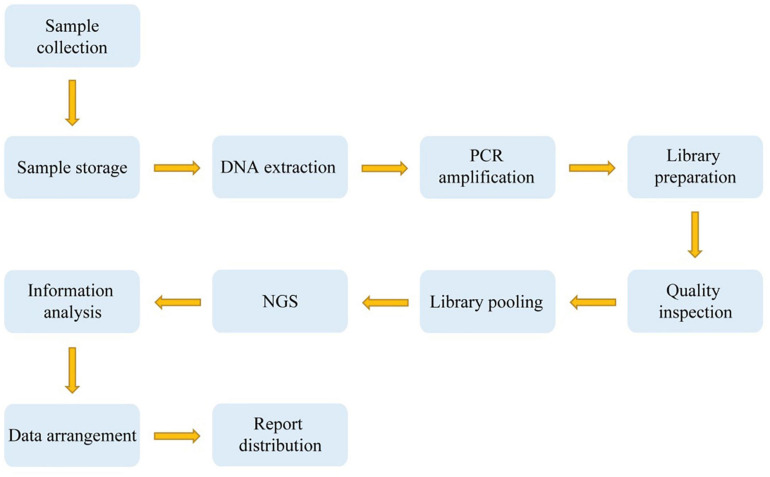
A technical route of deafness genetic screening.

We then carried out quality inspection and pooling of the library. The DNA double strands were thermally denatured into single strands after pooling, followed by the addition of the cyclic buffer and ligase to prepare circular DNA according to the cyclization reaction. The circular DNA molecules were used to make DNA nanoballs (DNBs) by rolling-circle replication (RCR). The concentration of DNBs was determined on a Qubit^®^ 2.0 fluorometer using the QubitTM ssDNA Assay kit (Thermo Fisher Scientific, Waltham, MA, United States), and a DNB concentration within the range of 8–40 ng/μL was considered ideal. The DNBs were loaded onto chips and sequenced on the MGISEQ-2000 sequencing platform (BGI, Shenzhen, China). Figure 1 indicates the overall technical route.

### Bioinformatics

The align sequence reading was compared with the human reference genome (hg19) and variants were called using the GATK software package and a localized database based on Human Gene Mutation Database, 2020. Further annotation was obtained using data from Human Gene Mutation Database,^1^ OMIM,^2^ PubMed,^3^ GeneReviews,^4^ dbSNP,^5^ 1000 Genomes Project,^6^ the National Heart, Lung, and Blood Institute Exome Sequencing Project,^7^ the National Center for Biotechnology Information Reference Sequence Database,^8^ and the UCSC genome browser,^9^ as well hearing loss-specific databases such as the Hereditary Hearing Loss Homepage.^10^

### Clinical Report

For the positive samples detected by high-throughput sequencing, mass spectrometry or Sanger sequencing would be used for secondary verification, and the results of these two detection methods should be compared. If the results were consistent, these two detection results would be written into the reports. When the results of the two methods were inconsistent, we arranged high-throughput sequencing and Sanger sequencing for re-verification and wrote a report based on the results of these two rounds of testing (Figure 1).

### Statistical Analysis

All data is statistically analyzed using SPSS 20.0 software. Pearson chi-square test was used for comparison among the samples. The 95% confidence interval of frequency of genes was calculated. *P* < 0.05 was considered statistically significant.

## Results

### General Clinical Data

A total of 5,120 neonatal babies were enrolled in the study, including 2,640 male (51.56%) and 2,480 female (48.44%) participants. In these cases, 2,611 mothers were primiparas (51%) and 2,509 mothers were nulliparous women (49%); 4,570 full term (89.26%), 264 premature delivery (5.16%), and 286 overdue delivery (5.58%); 2,974 natural delivery (58.09%) and 2,146 cesarean sections (41.91%). As Table 1 showed, 792 newborns (15.47%) were born with high-risk factors by neonatal screening. The highest proportion that occurred was hyperbilirubinemia, followed by premature delivery (Table 1).

**TABLE 1:** Main risk factors on birth.

**Risk factors**	**Cases (frequency, %)**
Hyperbilirubinemia	259 (5.06)
Premature delivery	146 (2.85)
Intrauterine infection	117 (2.29)
Umbilical cord around the neck	112 (2.19)
Maternal diabetes	92 (1.80)
Asphyxia neonatorum	33 (0.64)
Intrauterine distress	29 (0.56)
Deformity	20 (0.39)
Hypothyroidism	20 (0.39)
Maternal hypertension and preeclampsia	16 (0.31)
Respiratory distress	11 (0.21)
Light of birth weight	7 (0.14)
History of maternal syphilis	7 (0.14)
History of maternal Hepatitis B	6 (0.12)
Hypoxic–ischemic encephalopathy	5 (0.10)
Bacterial meningitis	2 (0.04)

### Outcomes of Hearing Screening

Of the 5,120 newborns, 227 neonates (4.43%) were referred bilaterally or unilaterally for the initial screening, and seven subsequent neonates (0.14%) failed in repeat screening at the age of 3 and 6 months. Three neonates (4.51%) were positive for genetic screening. At follow-up at 32 months, one case received a cochlear implant with profound hearing loss at one year, but he had not carried the common deafness genes. Three cases had normal hearing speech (one case had speech retardation). one case with homozygous variant c.[235del] in *GJB2* was referred bilaterally for hearing screening and developed moderate hearing loss, but her linguistic competence was normal. Two cases who were referred for hearing screening for two steps had normal linguistic competence, carried with c.[235del] heterozygote in *GJB2* and c.920C > T heterozygote in *SLC26A4.*

### Results of Genetic Screening

In total, 446 (8.71%) of the 5,120 newborns carried at least one genetic deafness-associated variant. Table 2 shows the 95% confidence interval of frequency of genes (Table 2). The probability of the genetic variants occurring in these newborns was as follows.

**TABLE 2:** Results of genetic screening.

**Genes**	**Cases (*N*)**	**Frequency ‰**	**95% CI**
*GJB2*	278	54.30	48.09–60.51
*GJB3*	30	5.86	3.77–7.95
*SLC26A4*	98	19.14	15.39–22.90
*MT-RNR1* (*12SrRNA*)	50	9.77	7.07–12.46
*TMC1*	1	0.20	0.00–0.58

1. For *GJB2* gene: 271 heterozygotes (5.29%) and 7 homozygotes, among which nine cases (3.24%, 9/278) carried pathogenic deafness-associated variants, including seven homozygotes and another two cases with two variants identified (phase unknown) (c.[109G > A] + [299_300delAT] and c.[235delC] + [257C > G]) (Table 3).
2. For *GJB3* gene: 30 heterozygotes (0.59%), including 16 c.[538C > T] and 14 c.[547G > A]. No cases carried pathogenic variants.
3. For *SLC26A4* gene: 96 heterozygotes (1.88%) and 2 homozygotes (0.04%). The 2 homozygotes were identified to carry pathogenic alleles (homozygotes, c.[1229C > T] + [1229C > T], c.[2168A > G] + [2168A > G]) (Table 4).
4. For *MT-RNR1*: 26 heteroplasmic variants (0.51%) and 24 homoplasmic variants (0.47%) including m.[1555A > G], m.[961T > C], and m.[1095T > C] (Table 5).
5. For *TMC1*: only one case was c.100C > T heterozygotes carrier. Followed-up by 30 months, the baby had normal hearing and speech.
6. There were 11 multilocus heterozygous variants of more than one gene. (0.22%, 11/5,120). All children with these two genes have passed hearing screening (Table 6).

**TABLE 3:** 278 *GJB2* variants and genotypes.

**Variants**	**Type**	**Cases (frequency, %)**
c.[235delC]	Heterozygote	111 (39.93)
c.[109G > A]	Heterozygote	109 (39.21)
c.[299_300delAT]	Heterozygote	22 (7.91)
c.[187G > T]	Heterozygote	6 (2.16)
c.[109G > A] + [109G > A]	**Homozygote**	5 (1.80)
c.[139G > T]	Heterozygote	4 (1.44)
c.[257C > G]	Heterozygote	3 (1.08)
c.[508_511dupAACG]	Heterozygote	3 (1.08)
c.[235delC] + [235delC]	**Homozygote**	2 (0.72)
c.[176_191delGCAAGAACGTGTG]	Heterozygote	2 (0.72)
c.[9G > A]	Heterozygote	1 (0.36)
c.[35dupG]	Heterozygote	1 (0.36)
c.[35G > A]	Heterozygote	1 (0.36)
c.[109G > A] + [299_300delAT]	Two variants identified, phase unknown	1 (0.36)
c.[134G > A]	Heterozygote	1 (0.36
c.[176_191delGCTGCAAGAACGTG]	Heterozygote	1 (0.36)
c.[176_191delGCTGCAAGAACG TGTG]	Heterozygote	1 (0.36)
c.[230G > A]	Heterozygote	1 (0.36)
c.[235delC] + [257C > G]	Two variants identified, phase unknown	1 (0.36)
c.[416G > A]	Heterozygote	1 (0.36)
c.[427C > T]	Heterozygote	1 (0.36)

*The bold means the pathogenic deafness-associated variants.*

**TABLE 4:** 98 *SLC26A4* variants and genotypes.

**Variants**	**Type**	**Cases (frequency, %)**
c.[919-2A > G]	Heterozygote	56 (57.14%)
c.[2168A > G]	Heterozygote	10 (10.20)
c.[1804-6G > A]	Heterozygote	8 (8.16)
c.[1174A > T]	Heterozygote	3 (3.06)
c.[1229C > T]	Heterozygote	3 (3.06)
c.[1975G > C]	Heterozygote	3 (3.06)
c.[1226G > A]	Heterozygote	2 (2.04)
c.[387delC]	Heterozygote	2 (2.04)
c.[920C > T]	Heterozygote	2 (2.04)
c.[1229C > T] + [1229C > T]	**Homozygote**	1 (1.02)
c.[1264-12T > A]	Heterozygote	1 (1.02)
c.[1336C > T]	Heterozygote	1 (1.02)
c.[1343C > T]	Heterozygote	1 (1.02)
c.[1707 + 5G > A]	Heterozygote	1 (1.02
c.[2168A > G] + [2168A > G]	**Homozygote**	1 (1.02
c.[439A > G]	Heterozygote	1 (1.02)
c.[589G > A]	Heterozygote	1 (1.02)
c.[754T > C]	Heterozygote	1 (1.02)

*The bold means the pathogenic deafness-associated variants.*

**TABLE 5:** Fifty *MT-RNR1* variants and genotypes.

**Variants**	**Type**	**Cases (frequency, %)**
m. [961T > C]	Heteroplasmic	22 (44.00)
m. [1555A > G]	Homoplasmic	17 (34.00)
m. [961T > C]	Homoplasmic	3 (6.00)
m. [1555A > G]	Heteroplasmic	3 (6.00)
m. [1095T > C]	Homoplasmic	4 (8.00)
m. [1095T > C]	Heteroplasmic	1 (2.00)

**TABLE 6:** Multilocus variation.

**Variants**	**Cases (frequency, %)**
*GJB3* c.[538C > T] + *GJB2* c.[109G>A]	2 (18.18)
*GJB3* c.[547G > A] + *GJB2* c.[176_191delGCAAGAACGTGTG]	1 (9.09)
*GJB2* c.[109G > A] + *SLC26A4* c.[754T > C]	1 (9.09)
*GJB2* c.[109G > A] + *SLC26A4* c.[919-2A > G]	1 (9.09)
*GJB2* c.[235delC] + *SLC26A4* c.[1804-6G > A]	1 (9.09)
*GJB2* c.[109G > A] + *12S rRNA* m.1095T > C	1 (9.09)
*GJB2* c.[299_300delAT] + *12S rRNA* m.961T > C	1 (9.09)
*GJB3* c.[538C > T] + *12S rRNA* m.1095T > C	1 (9.09)
*GJB3* c.[547G > A] + *SLC26A4* c.[919-2A > G]	1 (9.09)
*SLC26A4* c.[919-2A > G] + *12S rRNA* m.1555A > G	1 (9.09)

### Comparison of Positive Rate in Deafness Genes

Compared with 20 variants in the four genes of traditional testing, the positive rate of expanded screening in 159 variants of 22 deafness related genes increased significantly (65.2% on average), including *GJB2* increasing by 97.2%, *SLC26A4* by 21%, and *MT-RNR1* by 150%. It is suggesting that it was helpful to improve the clinical efficacy of deafness-related gene screening by increasing the scope of screening (Table 7).

**TABLE 7:** Comparison of genetic variants by expanded and conventional screening.

**Genes**	**159 Loci**	**20 Loci**	**Increase (%)**
*GJB2*	278(5.43%)	141(2.75%)	97.2
*GJB3*	30(0.59%)	30(0.59%)	0.0
*SLC26A4*	98(1.91%)	81(1.58%)	21
*MT-RNR1*	50(0.98%)	20(0.39%)	150
*TMC1*	1(0.02%)	0(0.00%)	
Totality	446(8.71%)	270(5.27%)	65.2

### Comparison of the Results and Data From Literature and gnomAD

By comparing present literature and gnomAD available data we found that: (1) Among the *GJB2* variants, the incidence rate of c.[235delC] was the highest, followed by c.[299_300delAT]. There were 115 cases with c.[109G > A], accounting for 2.25%, which was significantly lower than 8.35% in the East Asian population. (2) Among the *SLC26A4* variants, the incidence rate of c.[919-2A > G] was the highest, which was significantly higher than that in other regions. There were 7 cases with c.[1804-6G > A], accounting for 0.16%, which was lower than 0.29% of the East Asian population. (3) Among the *GJB3* variants, the incidence rate of the two hot spot variants (c.[538C > T] and c.[547G > A]) was higher than that of the literature reports. (4) Among the *MT-RNR1* variants, the incidence rate of m.[1555A > G] was more than data in China (Wang et al., 2019). All of them had statistical differences by one-way ANOVA (*P* < 0.05) (Table 8).

**TABLE 8:** The frequency of the pathogenic/likely pathogenic variants (more than four loci) in this study.

**Genes**	**Nucleotide Nomenclature**	**Allele frequency (‰)**					
		**This study**	**Study of Wang et al., 2019**	**East Asian from gnomAD**	**European (Finnish) from gnomAD**	**Ashkenazi Jewish from gnomAD**	**Latino from gnomAD**
*GJB2*	c.109G > A	224.61	N/A	834.50**	16.73**	80.26**	26.81**
	c.235delC	222.66	91.07**	65.15**	0	0	0
	c.299_300delAT	44.92	22.06*	9.02**	0	0	0
	c.187G > T	11.72	N/A	3.01	0	0	0
	c. 139G > T	7.81	N/A	0.50	0	0	3.67
	c.176_191del16	7.81	5.32	1.63	0	0	0
	c.257C > G	7.81	N/A	0.54	0	0	0
*SLC26A4*	c.919-2A > G	109.38	60.94**	50.64**	0	0	0
	c.2168A > G	21.48	11.00	16.04	0	0	0
	c.1804 -6G > A	15.63	N/A	29.10*	0	0	0.57
	c.1229C > T	7.81	5.50	3.51	1.19	0.97	1.13
*GJB3*	c.538C > T	31.25	12.67**	11.03**	0	0	0
	c.547G > A	27.34	4.94**	5.01**	0	0	43.74*
*MT-RNR1*	m.961T > C	48.83	N/A	N/A	N/A	N/A	N/A
	m.1555A > G	39.06	19.34**	N/A	N/A	N/A	N/A
	m.1095T > C	9.76	N/A	N/A	N/A	N/A	N/A

The frequency of the gene variants versus that in this study. *P < 0.05. **P < 0.01. GnomAD: https://gnomad.broadinstitute.org/

### Data of High-Risk Variants Detected in the Newborns

**FIGURE 2:**
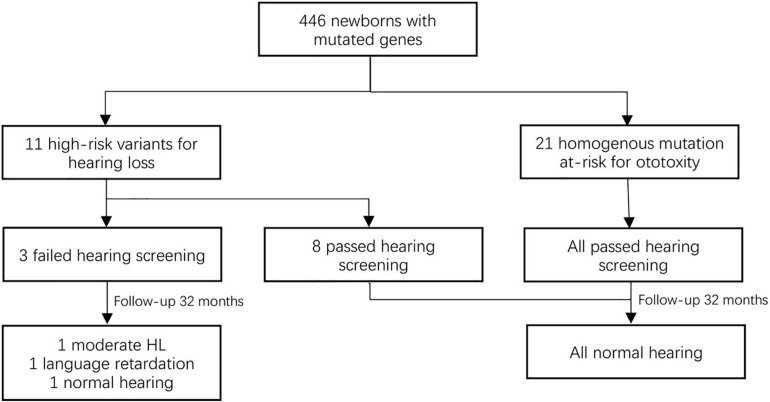
The hearing outcomes of newborns with positive genotypes.

Subsequently, we focused on the high-risk pathogenic variants in 11 newborns (0.21%, 11/5,120). Among these genes, nine cases (homozygotes and complex heterozygotes) in *GJB2* and 2 homozygotes in *SLC26A4* were identified. Though most of them (72.72%, 8/11) have passed hearing screening, further follow-up and a diagnostic audiological test are crucial methods for delayed hearing disorder. Twenty-four cases were homoplasmic variants in *MT-RNR1* who had a risk of deafness induced by ototoxic drugs, also had high-risk factors of pregnancy (Figure 2).

### Comparison of Genetic Variants in Mountain and Island Areas

In order to comprehensively evaluate the regional characteristics of deafness-related variants in different regions of Zhejiang province, we analyzed genetic variants between the remote mountain areas (971 cases) and island areas (500 cases) and found no statistical differences in positive rates of *GJB2, GJB3, SLC26A4*, and *MT-RNR1* genes, *P* > 0.05.

**FIGURE 3:**
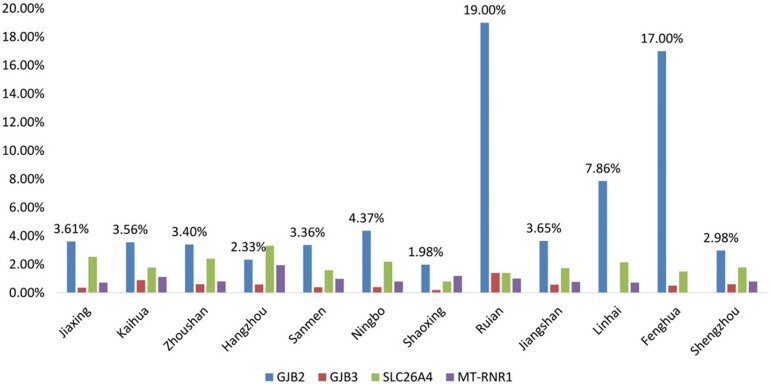
Comparison of the positive rates of genetic screening in 12 regional hospitals of Zhejiang Province.

The positive rates of *GJB3*, *SLC26A4*, and *MT-RNR1* were not statistically different in every region. However, the frequency of *GJB2* showed a statistical difference in every region, *P* < 0.05. Interestingly, it was significantly higher in Riuan and Fenghua (Figure 3).

### Comparison of Genetic Variants in High-Risk Pregnancies

The criteria for high-risk pregnancies are: (1) Pregnancy-related complications during pregnancy, or complications of chronic diseases such as hypertension and diabetes, (2) Mother-related complications during childbirth, (3) Premature expired or twin (multiple) pregnancies, and (4) Perinatal complications or malformations in newborns.

There were high-risk factors in 792 newborns (15.5%). The positive rate of the variant in *MT-RNR1* was significantly higher in neonates with maternal pregnancy risk factors, *P* < 0.05. There was no significant difference in no high-risk factors, hyperbilirubinemia, and non-hyperbilirubinemia.

**FIGURE 4:**
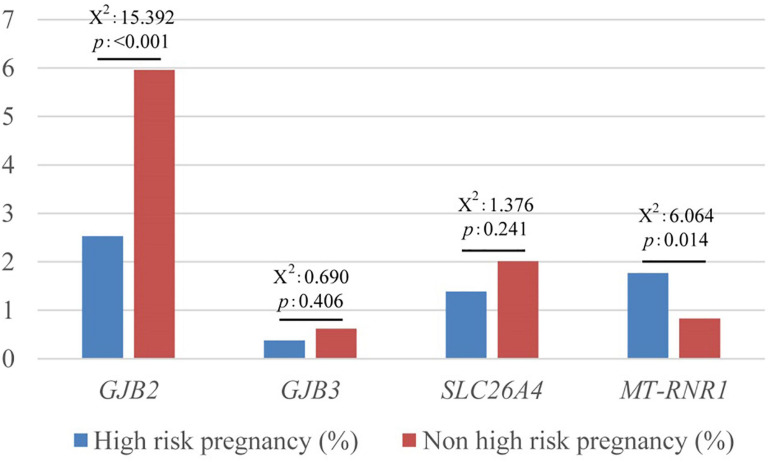
Comparison of high-risk and non-high-risk pregnancy in deafness gene mutations.

The positive rate of the variant in *GJB2* was significantly lower in newborns with high-risk factors for maternal pregnancy, *P* < 0.001, especially those with non-hyperbilirubinemia (x^2^ = 15.416, *P* < 0.001) (Figure 4).

## Discussion

Since Wang et al. (2011) first conducted multi-center large-scale neonatal hearing and deafness genetic screening in China in 2011, a large number of related studies have also been carried out in South China, Southwest China, North China, Taiwan, and other regions of China to provide abundant epidemiological data concerning the distribution and frequency of major genetic variants in China (Chu et al., 2015; Chen et al., 2019; Dai et al., 2019; Guo et al., 2020).

According to our study, of all the 446 newborns with positive deafness genes, 176 cases carried 20 loci in four genes, and more disease-causing variants have been detected. The overall positive rate increased by 65.2%. The positive rates of *GJB2*, *SLC26A4*, and *MT-RNR1* variants were increased by 97.2, 21, and 150%, respectively. Based on a meta-analysis conducted in China in 2019, there was a positive rate of 4.3% for 9 loci and 4.7% for 20 loci (Chen et al., 2019). Through expanded screening of deafness genes at 159 loci of 22 genes, the positive screening rate could be significantly increased.

Based on the study, high-risk pathogenic variants were found in 11 newborns (0.21%), most of them (90.91%) passed hearing screening. Further follow-up and diagnostic audiological tests are very important for a delayed hearing disorder.

### *GJB2* Variants and Genotypes

*GJB2* encodes gap junction protein Cx26 and has been shown to be the main cause of congenitally severe or profound sensorineural deafness. There are significant differences in frequency and variants among different races and populations (Jiang et al., 2016). In Caucasian people, more than 50% of *GJB2* pathogenic/likely pathogenic variants were found at c.35delG, about 70% were found in humans of Northern and Southern Europe, and at 167delT in German Jewish populations (Gasparini et al., 2000; Sugata et al., 2002). However, c.235delC is the most common variant in Asian populations (Kudo et al., 2001; Liu et al., 2002). The c.235delC could lead to frameshift variants and premature termination of Cx26 protein (Martínez et al., 2009). This study revealed c.235delC as the first mutant in *GJB2*, accounting for 40.74%, followed by c.299_300delAT, and the positive rate was higher than that in the East Asian population and gene pool data (Van Camp and Smith, 2020).

The results found that *GJB2* c.[109G > A] accounted for 2.25% of the total population, which was significantly lower than 8.35% in an East Asian population (Van Camp and Smith, 2020). This locus showed a significant difference in different regions and is rarely reported in the southeast coastal areas of China; while c.109G > A is frequently reported in patients with sensorineural deafness in the Hainan region of China (Huang et al., 2015). Homozygote or compound heterozygote in c.109G > A have been reported in a normal population, and whether this locus is pathogenic remains controversial (Chen et al., 2014). Xiao et al. (2017) have believed that c.109G > A might be related to delayed deafness. Previous cell experiments have proved that homozygote in c.109G > A can affect the adhesion between the expression product connexin, making it unable to form gap junction channels, leading to deafness. Thus, the variants might be related to mild to moderate deafness (Chen et al., 2016).

The positive rate of *GJB2* variants is found to be the highest in Ruian and Fenghua regions, suggesting that the rate of *GJB2* is high in certain regions.

In China, *GJB2* c.[235delC] + [235delC] homozygotes were the most common, which accounted for 55.10% of *GJB2* gene-related deafness, followed by 235delC heterozygote (20.40%), and 235delC/299-300delAT compound heterozygote (10.2%) (Zheng et al., 2000). The proportion of deafness caused by *GJB2* and common loci is different due to ethnic compositions and intermarriage within the ethnic groups in the northern and southern regions of Iran (Koohiyan et al., 2020). Some heterozygotes of *GJB2* can also cause deafness (Dai et al., 2019). *GJB2*c.551G > A (p. Arg184Gln) heterozygote is found in patients with deafness (Liu et al., 2020).

This study revealed two genetic patterns in *GJB2*, including *GJB3* c.[538C > T] + *GJB2* c.[109G > A] and *GJB3* c.[547G > A] + *GJB2* c.[176_191delGCAAGAACGTGTG]. Similar reports have shown *GJB2/GJB6* (Cheng et al., 2005) and *GJB2*/*GJB3* (Yuan et al., 2010) variants in 11% of deaf children, and genetic interaction between *GJB2* and *GJB3* in three unrelated families with autosomal recessive hereditary diseases (Liu et al., 2009). Therefore, the variant pattern with regard to hearing needs further follow-up study.

### *SLC26A4* Variants and Genotypes

The *SLC26A4* is located on chromosome 7q22.3 and is mainly expressed in the thyroid gland, inner ear, and kidney. The gene encodes Pendrin and is related to chlorine-iodine ion transportation (Scott et al., 1999). The variant leads to Pendred syndrome (PDS, congenital sensorineural deafness, cochlear abnormality, and goiter) and non-syndromic deafness with enlargement of the vestibular aqueduct (EVA). The main phenotype reported is prelingually or post-lingually progressive fluctuating hearing loss (Campbell et al., 2001).

Approximately 4–17% of deafness in patients in China is caused by *SLC26A4* variants, followed by the *GJB2* gene. The majority of the subjects (i.e., 81.81%) with EVA were detected with 919-2A > G (Qing et al., 2015). Up to now, 587 variants of *SLC26A4* have been found.^11^ c.919-2A > G is shown to be the most common variant of *SLC26A4* in the Chinese population, accounting for 57.63% of the total variants (Dai et al., 2020). This study found that c.919-2A > G has been the first to be observed, accounting for 56.99%, and the positive rate was higher than reported in Wang’s study and Southeast Asian data (Wang et al., 2019; Van Camp and Smith, 2020). *SLC26A4* c.[1804-6G > A] accounted for 0.16% of the total population, which is significantly lower than 0.29% (Van Camp and Smith, 2020) in East Asian populations. This reflects the regional characteristics of the *SLC26A4* variant spectrum in this region.

The two pathogenic sites of *SLC26A4* are c.[1229C > T] + [1229C > T] and c.[2168A > G] + [2168A > G]. The positive rate of these is similar to that of the gene pool (Van Camp and Smith, 2020). It has been found that *SLC26A4, GJB2*, and *GJB3* have double gene inheritance (Van Camp and Smith, 2020). No studies have reported this information, and it should be explored further in future studies. It has been reported that EVA might also be caused by the heterozygote of *SLC26A4* and *FOXI1*, or double gene inheritance of *SLC26A4* and *KCNJ10*, which is the first example where double gene inheritance has been proven to be the cause of deafness in human beings (Naseri et al., 2018).

### MT-RNR1 Variants and Genotypes

Variants in the mitochondrial gene *MT-RNR1* can lead to genetic susceptibility to aminoglycoside-induced hearing loss. At present, it is believed that a homoplasmic variant of the mtDNA*12SrRNA* gene is closely related to aminoglycoside antibiotic-induced deafness. Aminoglycoside drugs can accumulate in the cochlea and the vestibule and combine with *12SrRNA*, resulting in cell damage, apoptosis, and hearing loss. Previous studies have found that the *MT-RNR1*genotype demonstrated extensive heterogeneity, and *MT-RNR1* variants in different members of the same family can also have different penetrance rates. Some mutants carriers were also associated with mild to moderate sensorineural deafness without using aminoglycoside antibiotics (Barbarino et al., 2016). The variant modes are homozygotes and heterozygotes. The main genetic types include A155G, C1494T, T1095C, 961delC, A827G in *12SrRNA* and A7445G, G7444A, 7472insG, T7510C, and T7511C variants in *COI/tRNASer* (UCN) gene (Jacobs et al., 2005). According to previous Japanese studies, the carrying rate of m.1555A > G in sporadic neurosensorial hearing loss cases is about 3% (Mutai et al., 2017). The frequency in Africa is 2.4% (Tingang et al., 2019), and in eastern China it is 5.95%, which is significantly higher than that in other regions (Sun et al., 2019).

In this study, 21 cases are homoplasmic variants and are potentially sensitive to aminoglycoside antibiotics-induced deafness. The main locus is m.1555A > G accounted for 3.906‰ in the total sample, and the frequency is significantly higher than that reported in China, Europe, and Southeast Asia (Van Camp and Smith, 2020). The second is m.961T > C and m.1095T > C. Due to maternal inheritance of *MT-RNR1*, the variants are relatively stable in the population, suggesting a high incidence of *MT-RNR1* variants in this region. The positive rate of *MT-RNR1* variants is found to be significantly higher in newborns with high-risk pregnancy factors (*P* < 0.05). No relevant literature has reported its correlation.

### *GJB3* Variants and Genotypes

*GJB3* is located on the human chromosome lp33-35 and encodes connexin 31 with 270 amino acids. Its variant is closely related to the gap connexin 31 (eonnexin31, Cx31) variant encoded by *GJB3* (Xia et al., 1998). *GJB3* was first cloned by Chinese scientist Xia Jiahui in 1998. The variants at c.538C > T and c.547G > A sites lead to autosomal dominant non-syndromic deafness, mainly resulting in high-frequency hearing loss (Xia et al., 1998). More than 10 different variants were found in deaf patients of Spain (López-Bigas et al., 2001), Turkey (Uyguner et al., 2003), and China (Xia et al., 1998; Liu et al., 2000, 2009). However, the pathogenicity of *GJB3* is based only on reports with some cases and there is no available epidemiological incidence. Therefore, its pathogenicity is still controversial, and it is believed that it can lead to delayed deafness (Li et al., 2014). Two common disease-causing variants c.538C > T and c.547G > A were detected in this study, which is higher in proportion than that reported in the previous studies conducted in China and the data of the East Asian population (Van Camp and Smith, 2020). Under the condition that the pathogenicity of this gene cannot be excluded, it is necessary to strengthen the follow-up of this population.

In conclusion, the number of genes and loci for deafness screening was increased and the positive rate of screening is greatly improved in this study. Patients with genetic variants and normal hearing need to prolong the follow-up time for observing changes in hearing. Moreover, this study has defined the distribution range, genotypes, and pathogenic variants in Zhejiang province. It is expected to further expand the target range, more accurately and comprehensively analyze the spectrum and frequency of disease-causing variants, and establish a database of deafness gene variants carried with regional characteristics.

## Limitation

Even though this study involved in-depth screening of 159 variants in 22 deafness genes, it is impossible to cover all deafness-related variants or some new and rare loci. Genetic screening is not undertaken in all newborns in Zhejiang province. We will expand the screening population in the future and search for more regional characteristics of the pathogenic and likely pathogenic variants spectrum. For cases with high-risk deafness genes, it is necessary to further extend the follow-up time as later-onset hearing disorders may occur in.

## Data Availability Statement

The data presented in the study are deposited in the CNGB (db.cngb.org) repository, accession number CNP0001814.

## Ethics Statement

The studies involving human participants were reviewed and approved by Research Ethics Committee of The First Affliated Hospital, College of Medcine, Zhejiang University. Written informed consent to participate in this study was provided by the participants’ legal guardian/next of kin.

## Author Contributions

YL and LC were responsible for the collection, collation, and statistics of the data, the drawing of the figures and forms, and retrieving of the references. YL was responsible for the gene detection, analyzing, and discussion. YX was corresponding author and responsible for designing, organization, analyzing, and discussion. WC was responsible for part of audiological examinations. The other authors were responsible for the collection and collation of samples and data. All authors contributed to the article and approved the submitted version.

## Conflict of Interest

The authors declare that the research was conducted in the absence of any commercial or financial relationships that could be construed as a potential conflict of interest.

## Footnotes

1 http://www.hgmd.cf.ac.uk/ac/index.php

2 http://www.omim.org/

3 http://www.ncbi.nlm.nih.gov/pubmed

4 http://www.ncbi.nlm.nih.gov/books/NBK1116/

5 http://www.ncbi.nlm.nih.gov/SNP/

6 http://www.1000genomes.org/

7 http://evs.gs.washington.edu/EVS/

8 http://www.ncbi.nlm.nih.gov/refseq/

9 http://genome.ucsc.edu/

10 https://hereditaryhearingloss.org

11 http://www.hgmd.cf.ac.uk/ac/gene.php?gene=SLC26A4
