# Supplementary material for: Multi-Center in-Depth Screening of Neonatal Deafness Genes: Zhejiang, China
Source: Front Genet. 2021 Jul 2;12:637096. doi: 10.3389/fgene.2021.637096 (PMC8282931; doi:10.3389/fgene.2021.637096)
Supplement: Supplementary file 1 [file Table_1.docx]

Supplementary table. 22 genes and 159 variants

| **Number** | **Gene** | **Detected loci** |
| --- | --- | --- |
| 1 | *CDH23* | c.902G>A |
| 2 | *COL11A1* | c.4171-2A>G |
| 3 | *DFNA5* | c.1183+4A>G |
| 4 | *DFNB59* | c.547C>T |
| 5 | *DSPP* | c.52G>T |
| 6 | *GJB2* | c.109G>A, c.176_191delGCTGCAAGAACGTGTG, c.235delC, c.269dupT, c.280_284dupCACGT, c.290dupA, c.299_300delAT, c.310_323delAGGAAGTTCATCAA, c.535G>A, c.35delG, c.35dupG, c.504_505insAAGG, c.508_511dupAACG, c.509dupA, c.512_513insAACG, c.564_565delGA, c.575_576delCA, c.576delA, c.632_633delGT, c.99delT, c.104T>G, c.107T>C, c.132G>C, c.134G>A, c.167delT, c.175G>A, c.187G>T, c.224G>A, c.229T>C, c.250G>A, c.250G>C, c.257C>G, c.269T>C, c.283G>A, c.298C>T, c.34G>C, c.35G>A, c.35G>T, c.365A>T, c.427C>T, c.416G>A, c.439G>A, c.44A>C, c.487A>C, c.487A>G, c.506G>A, c.50C>T, c.313_326delAAGTTCATCAAGGG, c.596C>T, c.605G>T, c.9G>A, c.94C>T, c.95G>T, c.132G>A, c.139G>T, c.169C>T, c.230G>A, c.231G>A, c.238C>T, c.370C>T, c.598G>T, c.71G>A |
| 7 | *GJB3* | c.520G>A, c.547G>A, c.538C>T |
| 8 | *KCNJ10* | c.538C>T |
| 9 | *MT-RNR1* | m.1095T>C, m.1494C>T, m.1555A>G, m.961T>C |
| 10 | *MT-TL1* | m.3243A>G |
| 11 | *MT-TS1* | m.7445A>G |
| 12 | *MYO15A* | c.8183G>A, c.8767C>T |
| 13 | *MYO7A* | c.731G>C, c.1996C>T, c.2005C>T, c.700C>T, c.133-2A>G |
| 14 | *OTOF* | c.3624delG |
| 15 | *PCDH15* | c.1088delT, c.1036G>T |
| 16 | *SLC26A4* | c.281C>T, c.1181_1183delTCT, c.1198delT, c.1238delA, c.1341delG, c.1520delT, c.1547dupC, c.1555_1556delAA, c.1586delT, c.1692dupA, c.1746delG, c.349delC, c.365dupT, c.387delC, c.1264-12T>A, c.1707+5G>A, c.1804-6G>A, c.1151A>G, c.1160C>T, c.1173C>A, c.1174A>T, c.1226G>A, c.1229C>T, c.1334T>G, c.1343C>T, c.1540C>A, c.1541A>G, c.1586T>G, c.1594A>C, c.1975G>C, c.1997C>T, c.2015G>A, c.2027T>A, c.2162C>T, c.2168A>G, c.230A>T, c.259G>T, c.367C>T, c.439A>G, c.589G>A, c.626G>T, c.679G>C, c.707T>C, c.716T>A, c.754T>C, c.920C>T, c.1336C>T, c.1343C>A, c.1540C>T, c.1554G>A, c.170C>A, c.170C>G, c.1768A>T, c.249G>A, c.1615-1G>A, c.1615-2A>G, c.165-1G>A, c.919-2A>G, c.1341+1G>C, c.2089+1G>A, c.600+2T>A |
| 17 | *SOX10* | c.565G>T, c.621C>G |
| 18 | *TCOF1* | c.386_387delCA, c.422dupA, c.497_500delATAC |
| 19 | *TMC1* | c.100C>T |
| 20 | *USH1G* | c.84dupC, c.113G>A |
| 21 | *WFS1* | c.1511C>T, c.1433G>A |
| 22 | *WHRN* | c.1267C>T |

*The classification of variants and the corresponding color: Pathogenic, Likely pathogenic, VUS, Uncertain significance, Drug response
